# Supplementary material for: Enhancing single-cell transcriptomics using interposed anchor oligonucleotide sequences
Source: Commun Biol. 2025 Jan 16;8:67. doi: 10.1038/s42003-025-07474-5 (PMC11739374; doi:10.1038/s42003-025-07474-5)
Supplement: Supplementary file 2 — Supplementary Information [file 42003_2025_7474_MOESM2_ESM.pdf]

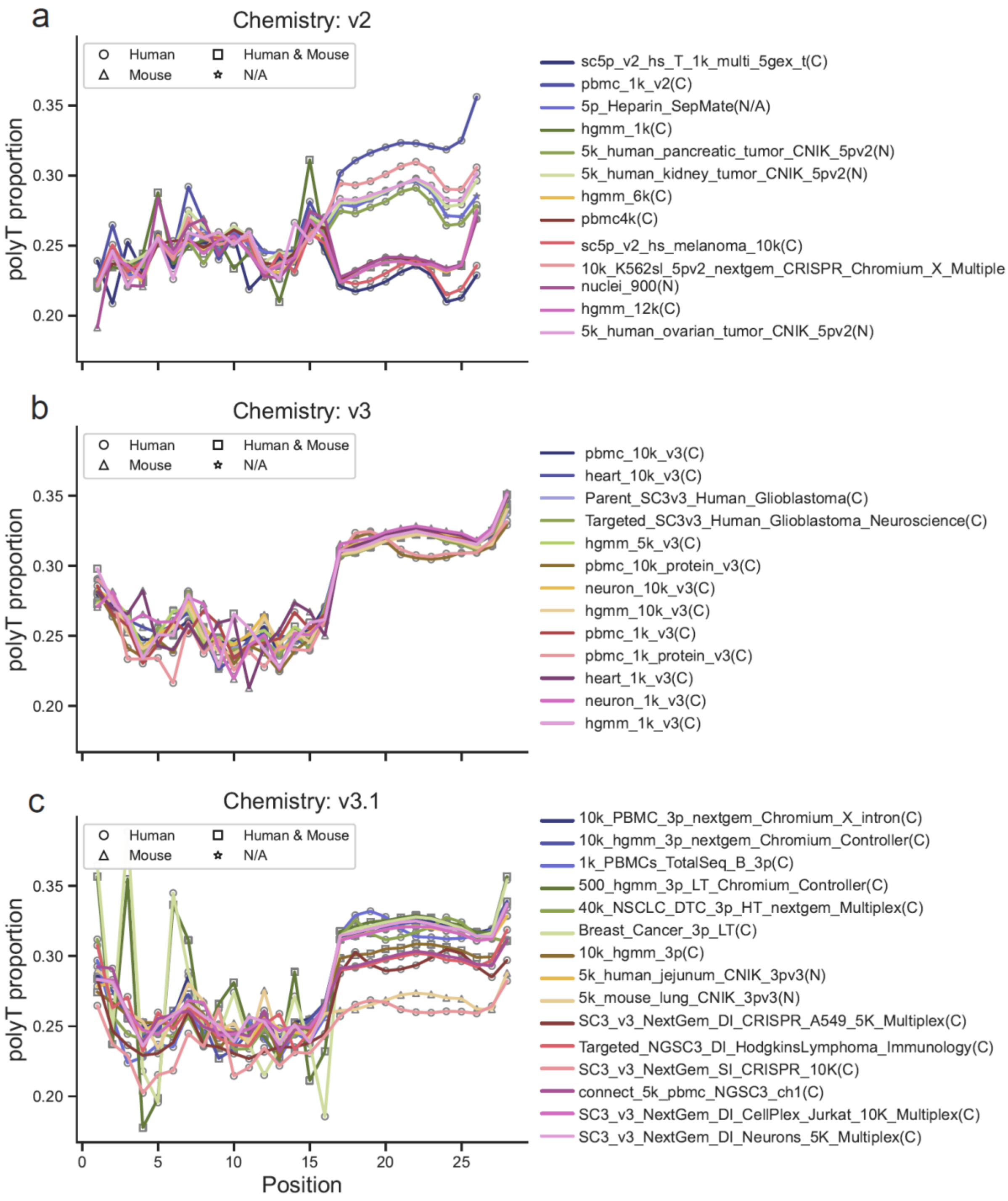

Supplementary Figure 1: Comparative Analysis of Thymine (T) Base Proportion in 10X Beads Across Versions. This figure delineates the frequency distribution of thymine (T) bases in two different versions of 10X beads. **a**) illustrates the prevalence of T bases in version 2 of the 10X beads, using data obtained from public datasets. **b**) represents the frequency of T bases in version 3 of the 10X beads. **c**) represents the frequency of T bases in version 3.1 (updates for version 3) of the 10X beads. This comparison includes a total of 41 datasets (**see Methods** for details) and aims to elucidate potential variations in base composition between the two types of bead versions. C: sampled from cells. N: sampled from nuclei. N/A: not available.

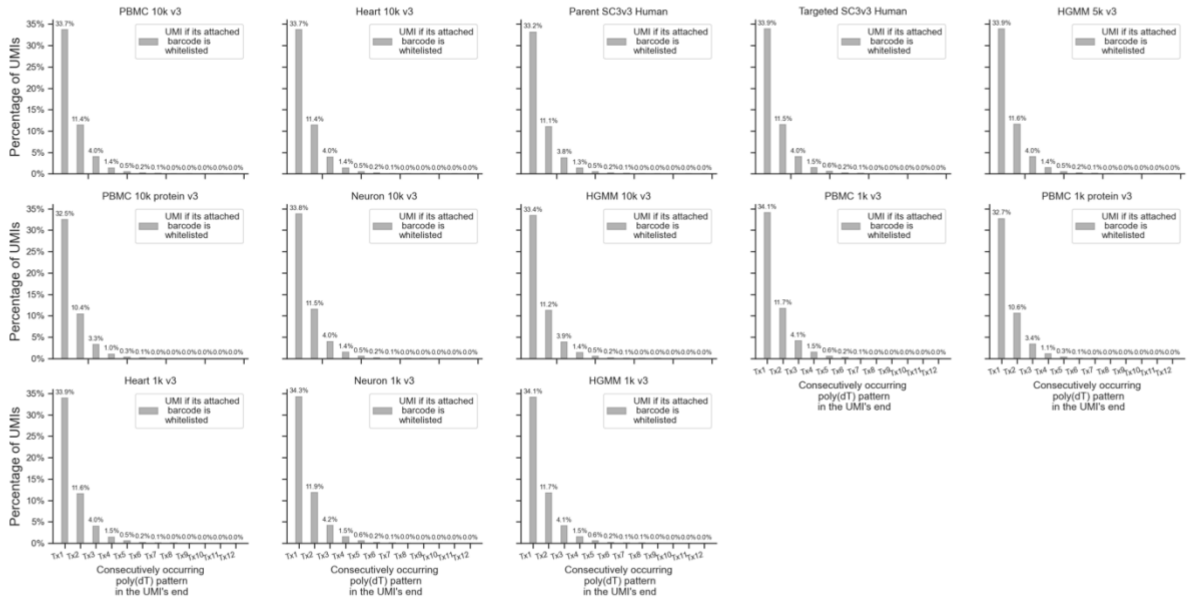

Supplementary Figure 2. Proportion of UMIs in chemistry V3 10X Chromium beads with terminal thymine (T) bases. The x-axis indicates the number of consecutive terminal T bases per UMI, categorized as 1xT (UMI ending in N...T), 2xT (N...TT), 3xT (N...TTT), etc. The y-axis displays the percentage of UMIs ending with each respective T-base configuration. This analysis quantifies the frequency of terminal T bases across UMIs, highlighting variations in nucleotide distributions.

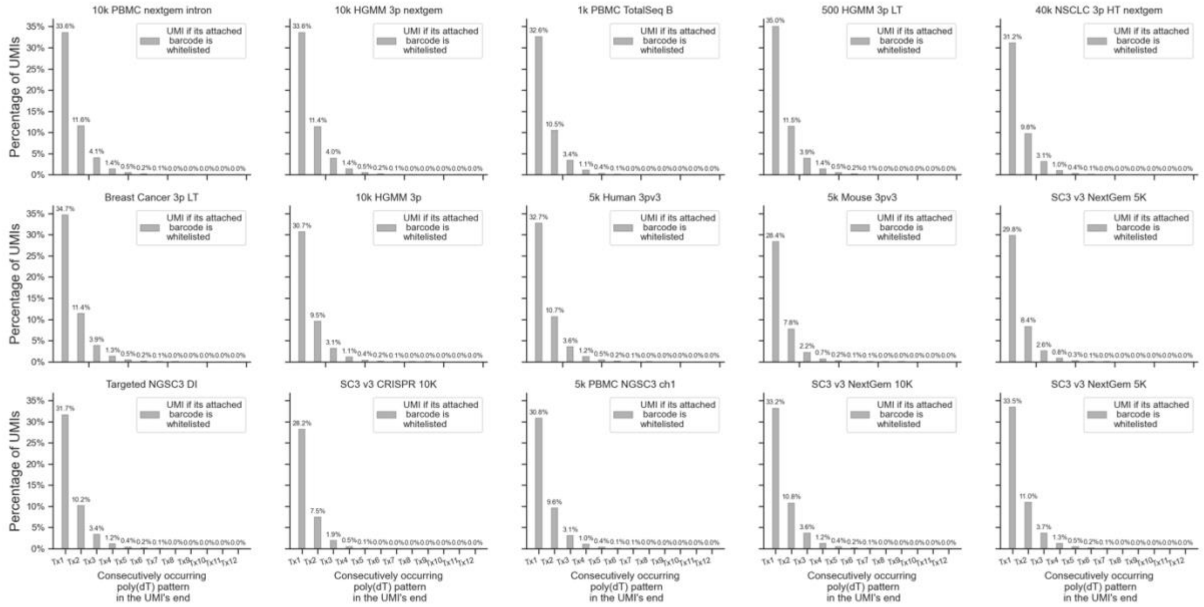

Supplementary Figure 3. Proportion of UMIs in chemistry V3.1 10X Chromium beads with terminal thymine (T) bases. The x-axis indicates the number of consecutive terminal T bases per UMI, categorized as 1xT (UMI ending in N...T), 2xT (N...TT), 3xT (N...TTT), etc. The y-axis displays the percentage of UMIs ending with each respective T-base configuration. This analysis quantifies the frequency of terminal T bases across UMIs, highlighting variations in nucleotide distributions.

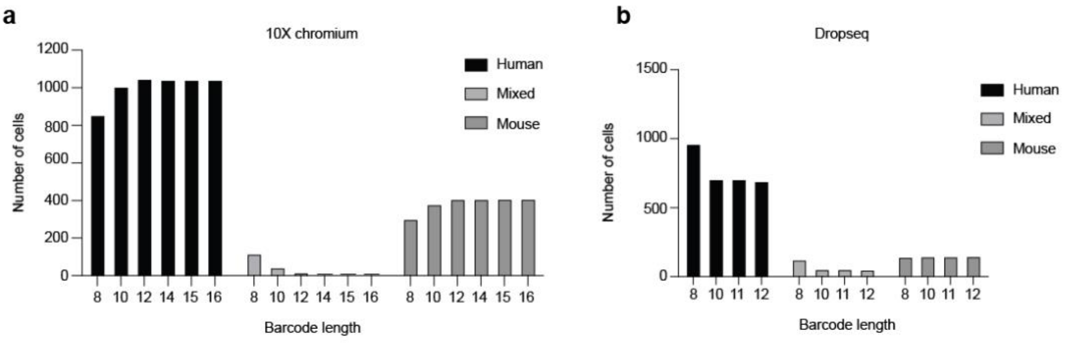

Supplementary Figure 4. Human and mouse cells were co-encapsulated using both the 10X Chromium device and Dropseq, under the assumption that compromised barcode assignment would lead to a higher incidence of mixed cells. The expectation was that barcode truncation would result in a noticeable increase in mixed cell counts. However, analysis of both 10X Chromium and Dropseq datasets showed no significant rise in mixed cells, suggesting that synthesis irregularities have minimal impact on barcode assignment.

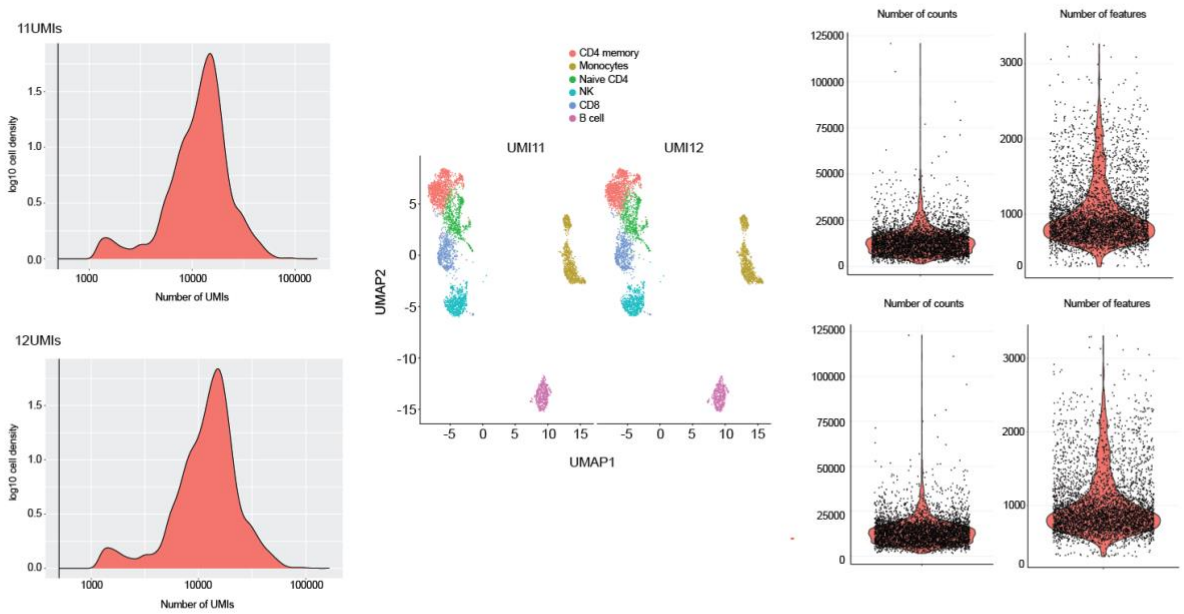

Supplementary Figure 5: 10X data showing quality plots showing the 5k 10X public data sequenced using ONT. The data plotted on the left shows the number of UMIs captured using 11and 12 UMI lengths. The middle plot shows the annotation of the cells and accompanies Fig. 2e and Fig. 2f. The right hand side shows the number of count and features for each cell plotted as a violin plot.

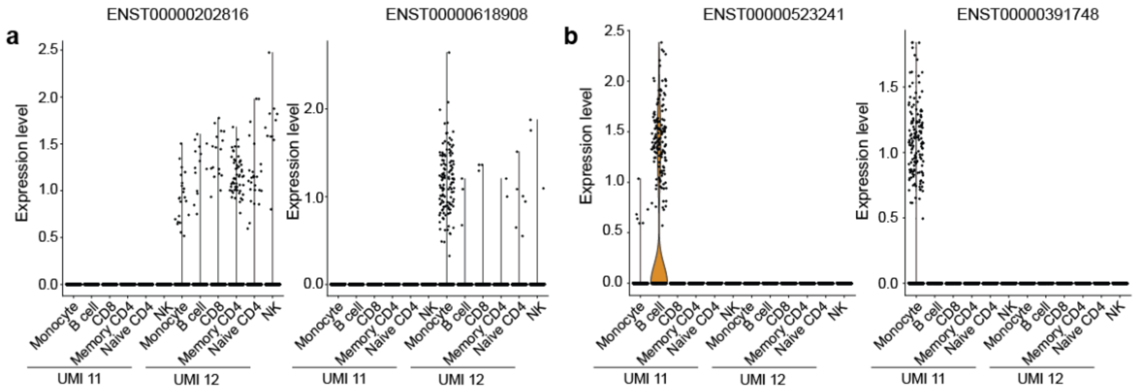

Supplementary Figure 6: a, Violin plots showing the expression of ENST00000202816 (ESF1) and ENST00000618908 (LAP3) across the cell annotated 10X Chromium 5k PBMC v3.1 dataset, analysed using UMI lengths of 11 or 12. **b,** Violin plots depicting the expression of ENST00000523241 (Paired Box 5; PAX5) and ENST00000391748 (Leukocyte immunoglobulin-like receptor subfamily B member 2; LILRB2) across the cells in the 10X Chromium 5k PBMC v3.1 dataset, offering significant into the significance of UMI length in gene expression analysis.

The violin plots serve to illustrate the distribution of gene expression levels for two genes, ESF1 and LAP3, across a spectrum of cell types utilising a Unique Molecular Identifier (UMI) of 12 nucleotides in length. The data indicates a broad expression profile for these genes under these conditions. In contrast, when the UMI length is reduced to 11 nucleotides, the previously detected gene expression is not observed. This pattern implicates the terminal variability of the UMI as a potential source of erroneous read assignment.

Conversely, the genes PAX5 and LILRB2, known to be predominantly expressed in B cells, exhibit a high level of expression when analysed with an 11-nucleotide UMI. This expression is not detected with a 12-nucleotide UMI, suggesting that in these instances, the terminal variability of the UMI is leading to a misclassification of reads. This could be due to the ineffective computational demultiplexing of data, where the critical nuance at the end of the UMI is not adequately accounted for, resulting in a loss of informative reads.

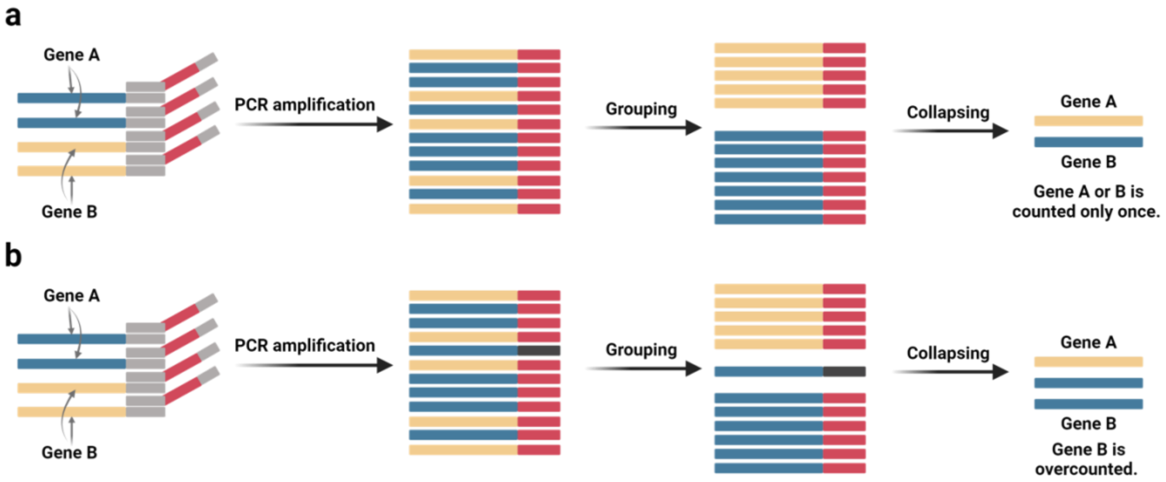

Supplementary Figure 7: Empirical evaluation of transcript counting with Common Molecular Identifiers (CMIs). **a**, An Ideal CMI collapsing example: In this scenario, transcripts (two blue and two green) are labelled with a common molecular identifier barcode (CMIs; labelled as red) and amplified via PCR. During transcripts grouping, all transcripts are labelled with the same common sequence. Therefore, following demultiplexing, each transcript should receive a count of one for every instance of detection. b, Increased counts result from the introduction of errors: This figure illustrates the effect of the errors within the CMI sequence. Any error introduced during PCR or sequencing creates a new CMI (labelled as yellow), resulting in an increase in transcript counts. This allows empirical evaluation of the effect of errors on the counting of transcripts, providing valuable insights into the accuracy of transcript quantification.

a

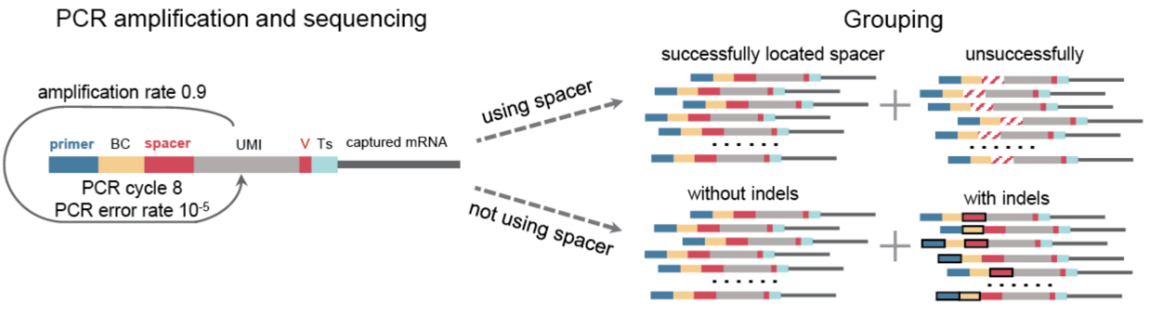

Supplementary Figure 8: Comparative Analysis of UMI Identification Efficacy with and without Anchor Utilisation. Illustrates the simulation workflow for assessing UMI (Unique Molecular Identifier) recognition efficiency employing an anchored versus non-anchored approach. Key features include the depiction of potential indels (insertions/deletions) or substitution errors within the anchor region (represented by white slashes) and other critical components such as primers, barcodes, or UMIs (denoted by dark squares). In simulation scenarios utilising an anchor, reads exhibiting errors in the anchor region are categorised as unsuccessful in UMI identification. Conversely, in the absence of an anchor, UMI identification success is determined by the direct analysis of the UMI sequence itself, with indels preceding the UMI indicating identification failure. Methodological details and the analytical framework underpinning these evaluations are elaborated within the Methods section.

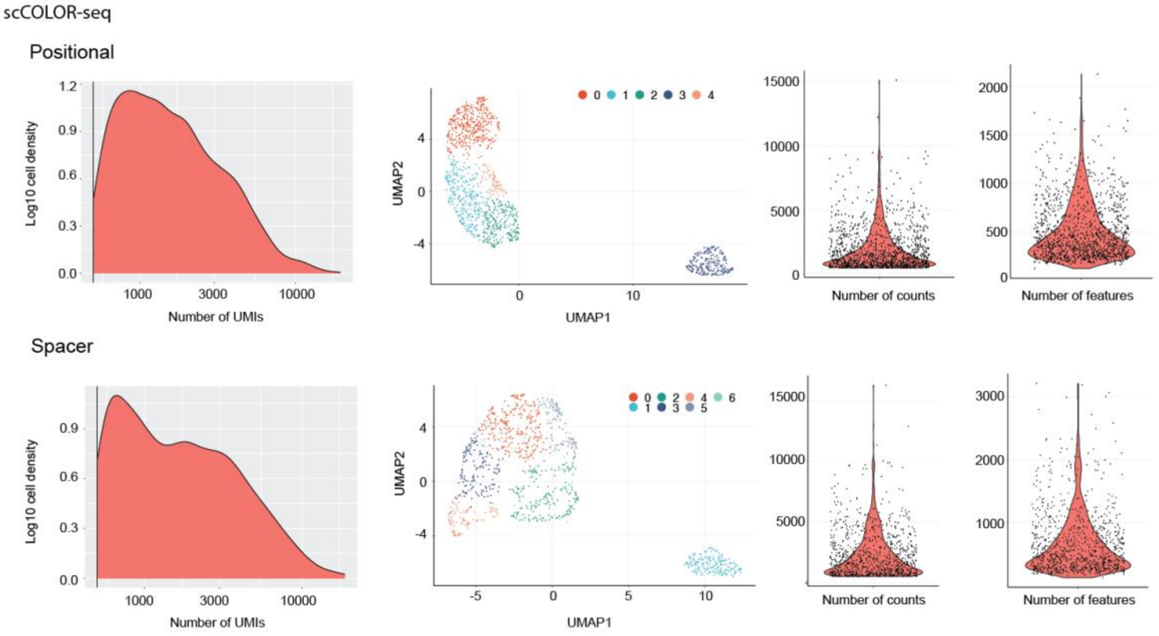

Supplementary Figure 9: scCOLOR-seq showing the quality metrics for the positional and anchor analysis approaches. The data plotted on the left shows the number of UMIs captured using positional and anchor approaches. The middle plot shows the annotation of the cells into clusters. The right-hand side shows the number of count and features for each cell plotted as a violin plot.

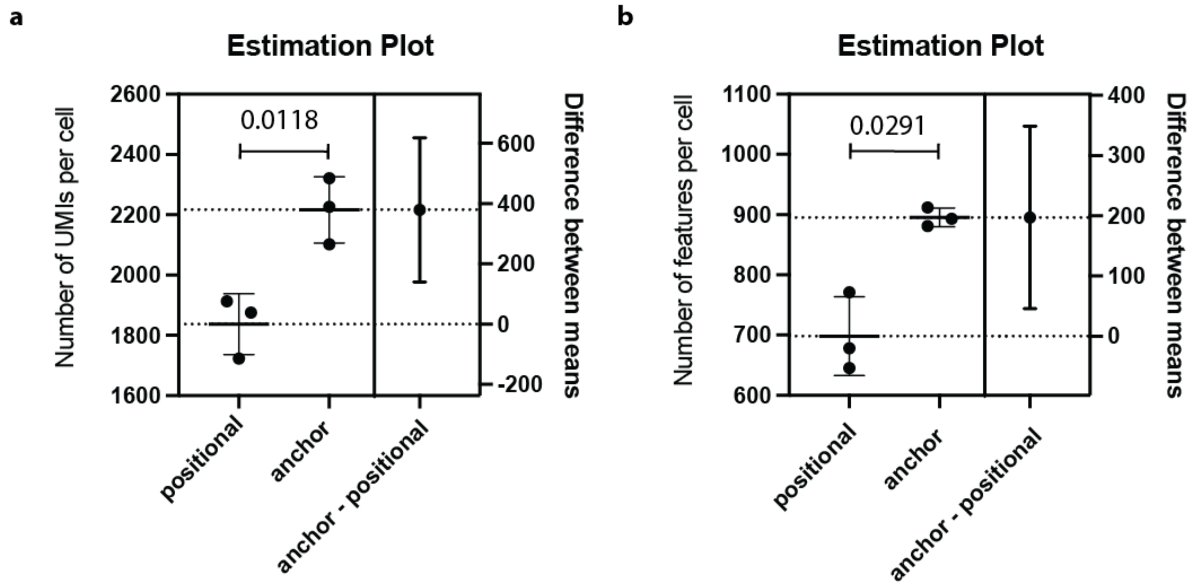

Supplementary Figure 10: The impact of positional and spacer strategies on UMI and feature detection in scCOLOR-seq using the positional and anchor strategy. **a**, shows the estimation plot of UMIs per cell, where the anchor strategy yields a significantly higher mean number of UMIs per cell compared to the positional strategy ($p = 0.0118$). **b**, presents the estimation plot of detected features (genes) per cell, with the anchor strategy also showing a higher mean number of features per cell relative to the positional strategy ($p = 0.0291$). In both panels, the right-side graphs depict the differences in means between the two strategies, with error bars representing the 95% confidence intervals. Statistical significance was determined using a t-test with Welch's correction.

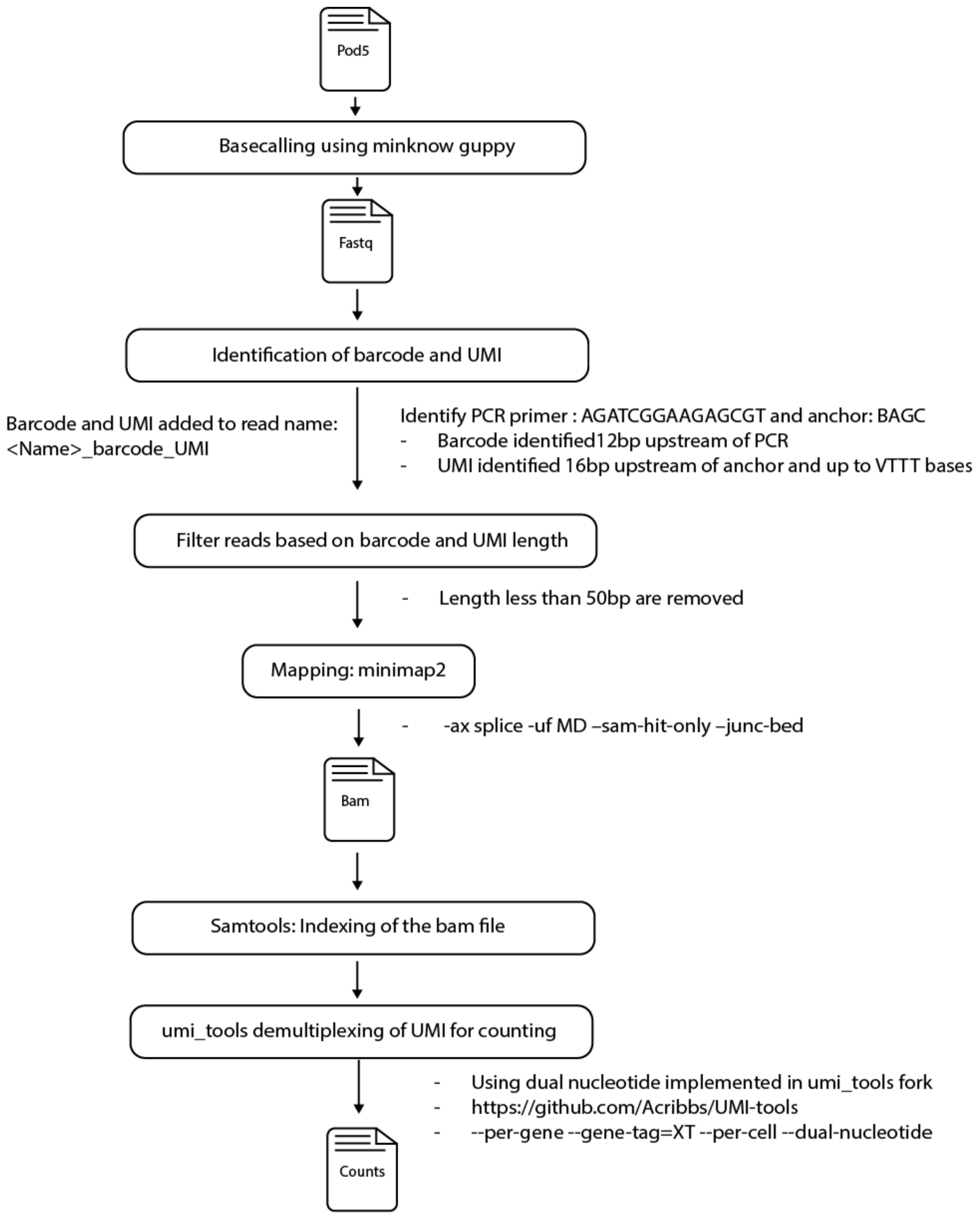

Supplementary Figure 11: A flow diagram showing the computational analysis steps involved in the analysis of scCOLOR-seq2 data.
