# Supplementary material for: Enhancing single-cell transcriptomics using interposed anchor oligonucleotide sequences
Source: Commun Biol. 2025 Jan 16;8:67. doi: 10.1038/s42003-025-07474-5 (PMC11739374; doi:10.1038/s42003-025-07474-5)
Supplement: Supplementary file 4 — Description of Additional Supplementary Files [file 42003_2025_7474_MOESM4_ESM.docx]

Description of Additional Supplementary Files

**File name:** Supplementary Data 1

**Description:** The differential expression analysis results as the evidence of truncation in the oligonucleotides synthesized on beads.

**File name:** Supplementary Data 2

**Description:** 10X datasets using Chemistry V2.

**File name:** Supplementary Data 3

**Description:** 10X datasets using Chemistry V3.

**File name:** Supplementary Data 4

**Description:** 10X datasets using Chemistry V3.1.

**File name:** Supplementary Data 5

**Description:** Data source for graphs in main figures.
